# Characterizing the Leaf Transcriptome of *Chrysanthemum rhombifolium* (Ling et C. Shih), a Drought Resistant, Endemic Plant From China

**DOI:** 10.3389/fgene.2021.625985

**Published:** 2021-02-11

**Authors:** Wenjie Zhang, Hongyuan Xu, Xiaxia Duan, Jing Hu, Jingjing Li, Liang Zhao, Yueping Ma

**Affiliations:** ^1^College of Life and Health Sciences, Northeastern University, Shenyang, China; ^2^College of Life Sciences, Northwest A&F University, Yangling, China

**Keywords:** Asteraceae, stress tolerance, ornamental plant, RNA-seq, SSR

## Abstract

*Chrysanthemum rhombifolium* (Ling et C. Shih), an endemic plant that is extremely well-adapted to harsh environments. However, little is known about its molecular biology of the plant's resistant traits against stress, or even its molecular biology of overall plant. To investigate the molecular biology of *C. rhombifolium* and mechanism of stress adaptation, we performed transcriptome sequencing of its leaves using an Illumina platform. A total of 130,891 unigenes were obtained, and 97,496 (~74.5%) unigenes were annotated in the public protein database. The similarity search indicated that 40,878 and 74,084 unigenes showed significant similarities to known proteins from NCBI non-redundant and Swissprot protein databases, respectively. Of these, 56,213 and 42,005 unigenes were assigned to the Gene Ontology (GO) database and Cluster of Orthologous Groups (COG), respectively, and 38,918 unigenes were mapped into five main categories, including 18 KEGG pathways. Metabolism was the largest category (23,128, 59.4%) among the main KEGG categories, suggesting active metabolic processes in *C. rhombifolium*. About 2,459 unigenes were annotated to have a role in defense mechanism or stress tolerance. Transcriptome analysis of *C. rhombifolium* revealed the presence of 12,925 microsatellites in 10,524 unigenes and mono, trip, and dinucleotides having higher polymorphism rates. The phylogenetic analysis based on *GME* gene among related species confirmed the reliability of the transcriptomic data. This work is the first genetic study of *C. rhombifolium* as a new plant resource of stress-tolerant genes. This large number of transcriptome sequences enabled us to comprehensively understand the basic genetics of *C. rhombifolium* and discover novel genes that will be helpful in the molecular improvement of chrysanthemums.

## Introduction

Chrysanthemum (*Chrysanthemum morifolium* (Ramat.)Tzvel.; Asteraceae) is among the most popular flowers in China, and the most important cut flowers in the world, having a great ornamental and economical value (Song et al., 2018; Su et al., 2019). However, the long-term artificial domestication of chrysanthemums often causes declines in their resistance to environmental stressors and adaptability (Da Silva, 2003; Chen et al., 2011, 2012; Song et al., 2014), thereby limiting their use in landscaping and industrial production. Therefore, the development of Chrysanthemum cultivars with increased environmental tolerance has always been a goal of breeders (Su et al., 2019). Many stress resistance traits, and corresponding stress resistance gene resources identified in the wild chrysanthemum species (Zhao et al., 2009; Lu et al., 2010; Li et al., 2013), have a great significance for the genetic improvement of chrysanthemum cultivars.

RNA sequencing (RNA-Seq) is a powerful tool for quantifying and analyzing different types of RNA molecules using deep-sequencing technologies (Wang et al., 2009). It provides us large-scale transcript data with high throughput, accuracy, sensitivity and reproducibility which enabled us to generate an unprecedented global view of the transcriptome of the species (Angeloni et al., 2011; Jain, 2011). RNA-seq has been widely used in plants, especially for some non-model species and some large and complex genomes, greatly accelerating the discovery of novel genes, understanding the complex tissue-specific expression patterns, and regulation networks in higher plants (Li and Dewey, 2011; Wang et al., 2014, 2017; Wu et al., 2016).

**Figure 1:**
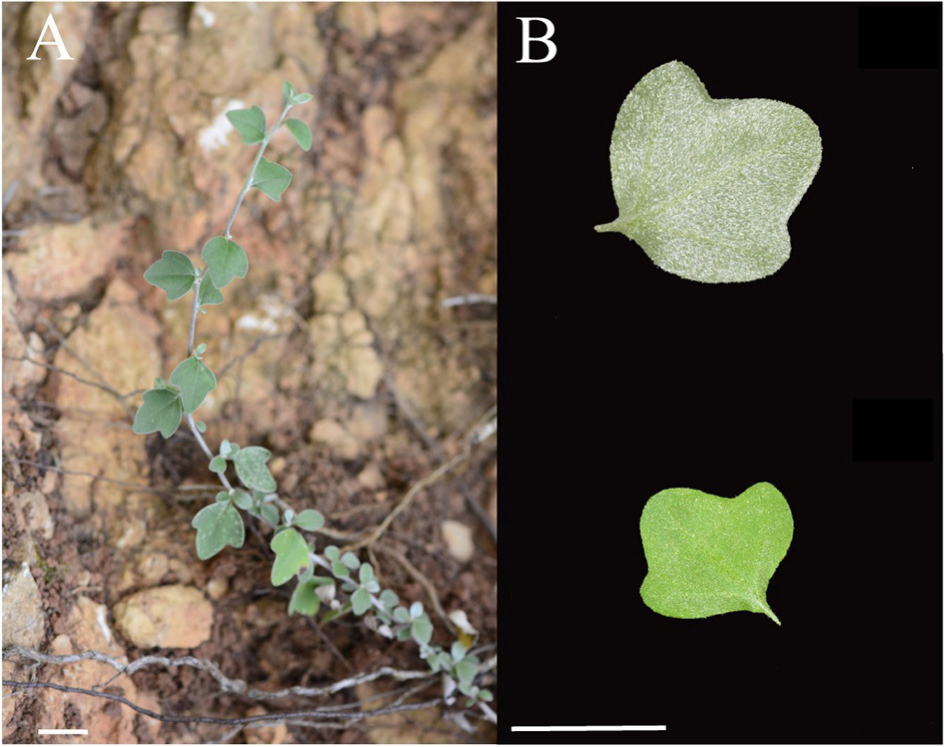
The plant of *C. rhombifolium*. **(A)** The habitat; **(B)** leaf, up, abaxially, down, adaxially. Scale bars = 2 cm.

*Chrysanthemum rhombifolium* Ling et Shih is a perennial herb endemic to Wushan, Chongqing in China (Shih and Fu, 1983; Bremer and Humphries, 1993) and has a high ornamental value. It has diamond-shaped leaves with dense abaxial pubescence and semi-lignified stems and branches (Figure 1). The species is well-adapted to environments characterized by high temperatures, low soil fertility, and drought (Zhao et al., 2009, 2010). However, few studies performed on *C. rhombifolium* except using as a sample in molecular phylogeny of *Chrysanthemum* (Masuda et al., 2009; Zhao et al., 2010; Li et al., 2014; Ma et al., 2020) or in geographical distribution of *Chrysanthemum* (Zhao et al., 2009; Li et al., 2013). Here, little is known about its molecular biology of overall plant or the plant's resistant traits against stress. This prompted us to characterize its leaf transcriptome using high-throughput RNA sequencing and *de novo* assembly to provide a comprehensive resource for understanding the biology of *C. rhombifolium* in general, and gain insights in improving the breeding of chrysanthemums and other related crops.

## Materials and Methods

### Plant Materials

We collected *C. rhombifolium* plants from Wushan of Chongqing in China and planted them in the Nurse Garden of the Northeastern University, China. Fresh, mature leaves were washed with sterile water, immediately frozen in liquid nitrogen, and stored at −80°C.

### RNA Isolation and cDNA Library Construction

Total RNA was isolated from the leaves using TRIzol reagent (Invitrogen^TM^ Life Technologies, CA, USA) following the manufacturer's instructions. The RNA quality was assessed using formaldehyde denaturing gel electrophoresis (28S:18S>2), a NanoPhotometer®spectrophotometer (IMPLEN, CA, USA), and RNA Nano 6000 Assay Kit of the Agilent Bioanalyzer 2100 system (Agilent Technologies, CA, USA). For RNA-Seq analysis, three biological replicates were used. Sequencing libraries were generated with 1 μg RNA sample using NEBNext® Ultra™ RNA Library Prep Kit for Illumina® (NEB, USA) following the manufacturer's recommendations. The mRNA was purified from total RNA using beads with Oligo (dT), and cut into short fragments with fragmentation buffer. First-strand cDNA was synthesized using random hexamer primers and M-MuLV Reverse Transcriptase (NEB, USA), and second-strand cDNA was synthesized using buffer, dNTPs, RNase H, and DNA polymerase I. The remaining overhangs were converted into blunt ends via exonuclease/polymerase activities. After adenylation of the 3′ ends of DNA fragments, NEBNext Adaptor with hairpin loop structure was ligated to prepare for hybridization. cDNA fragments, preferentially 250–300 bp in length, were selected by purifying the library fragments with AMPure XP system (Beckman Coulter, Beverly, USA). The size-selected, adaptor-ligated cDNA fragments were incubated with 3 μl USER Enzyme (NEB, USA) at 37°C for 15 min, followed by 5 min at 95°C before PCR. PCR was performed with Phusion High-Fidelity DNA polymerase, Universal PCR primers, and Index (X) Primer. The PCR products were purified (AMPure XP system) and library quality was assessed on the Agilent Bioanalyzer 2100 system.

### Sequencing and *de novo* Assembly

We sequenced the transcriptome library using the Illumina HiSeq 2500 platform, and generated paired-end reads. We filtered the raw data using the Filterfq program (BGI, Shenzhen, China) to remove adaptor sequences, reads in which unknown nucleotides (N) were >5%, and low-quality sequences with >20% low-quality bases (quality value ≦10) to generate clean data. The raw data were deposited in the Sequence Read Archive (SRA) of the National Center for Biotechnology Information (NCBI) with the Bioproject accession: PRJNA674029 and BioSample accessions:SAMN16633381- SAMN16633383.

We then used the Trinity software (v2.8.0; http://trinityrnaseq.sourceforge.net/) with default settings for *de novo* transcriptome assembly (Grabherr et al., 2011). Two contigs thus obtained were connected into a single scaffold to generate unigenes. These unigenes were further spliced to generate longer complete consensus sequences and to remove redundant sequences with TGICL (v 2.1; http://www.tigr.org/tdb/tgi/) (Pertea et al., 2003).

### Functional Annotation and Classification of Unigenes

We annotated the obtained unigenes using the NCBI Nr (non-redundant protein database), NCBI Nt (non-redundant nucleotide sequences), Swiss-Prot, Gene ontology terms (GO), and Protein family (Pfam) using BLAST 2 with an E-value cut-off of 10^−5^ to obtain information on protein function annotation. We also performed functional annotation using Clusters of Orthologous Groups of proteins (KOG/COG) and Kyoto Encyclopedia of Genes and Genomes (KEGG) databases to classify possible COG functions and KEGG pathways and predict possible functional classifications and molecular pathways, respectively (Conesa et al., 2005; Ye et al., 2006; Kanehisa et al., 2008).

### Phylogenetic Analysis

GDP-D-mannose 3′, 5′- epimerase (GME), regulates cell wall biosynthesis and ascorbate accumulation, playing an important role in plant development and abiotic stress tolerance (Tao et al., 2018). We extracted annotated GME unigene sequences, aligned them with other GME homologs retrieved from Genbank (http://www.ncbi.nlm.nih.gov/entyez/query.fcgi). Multiple alignments were made using MUSCLE (Edgar, 2004) in Geneious v.8.1.2 (http://www.geneious.com/; Kearse et al., 2012) and adjusted manually. We contructed the phylogenetic tree by the neighbor-joining (NJ) method with 1,000 bootstrap replicates using MEGA 7 (Kumar et al., 2016).

### Simple Sequence Repeats (SSRs) Prediction

We predicted SSR regions among all the assembled unigenes using MIcroSAtellite (MISA, http://pgrc.ipk-gatersleben.de/misa/; Zalapa et al., 2012). We detected the SSR motifs of mono-, di-, tri-, tetra-, penta-, and hexa-nucleotides with a minimum of twelve, six, five, five, four, and four repeats, respectively. For other parameters, default settings were used.

## Results and Discussion

### Illumina Paired-End Sequencing and Assembly

The 151,037,024 raw sequencing reads obtained from the Illumina sequencing were cleaned by removing low-quality data and adaptors, yielding 147,842,128 clean reads with Q20 bases at 97.9%, and a GC content of 44.44%. Using the overlapping information of high-quality reads from the Trinity software, 254,853 transcripts with an average length of 921 bp and N50 of 1,301 bp were generated. After that 130,891 unigenes with an average length of 807 bp and N50 of 1,034 bp were obtained (Table 1). The number and average length of the unigenes we obtained was larger and longer than the transcriptomes of the related species, *C. nankingense* (45,789) and *C. lavandulifolium* (108,737) (Wang et al., 2013, 2014), indicating the high quality of sequencing.

**Table 1:** Summary of sequence assembling of *C. rhombifolium* transcriptome.

**Category**		**Transcripts**	**Unigene**
Number	300–500 bp	102,216	59,296
	500-1 Kbp	74,883	41,353
	1 K-2 Kbp	53,959	21,862
	>2 Kbp	23,795	8,380
Total number		254,853	130,891
Mean length		921	807
N50		1,301	1,034
Total nucleotides		234,824,746	105,639,077

### Functional Annotation and Classification of All Non-redundant Unigenes

**Figure 2:**
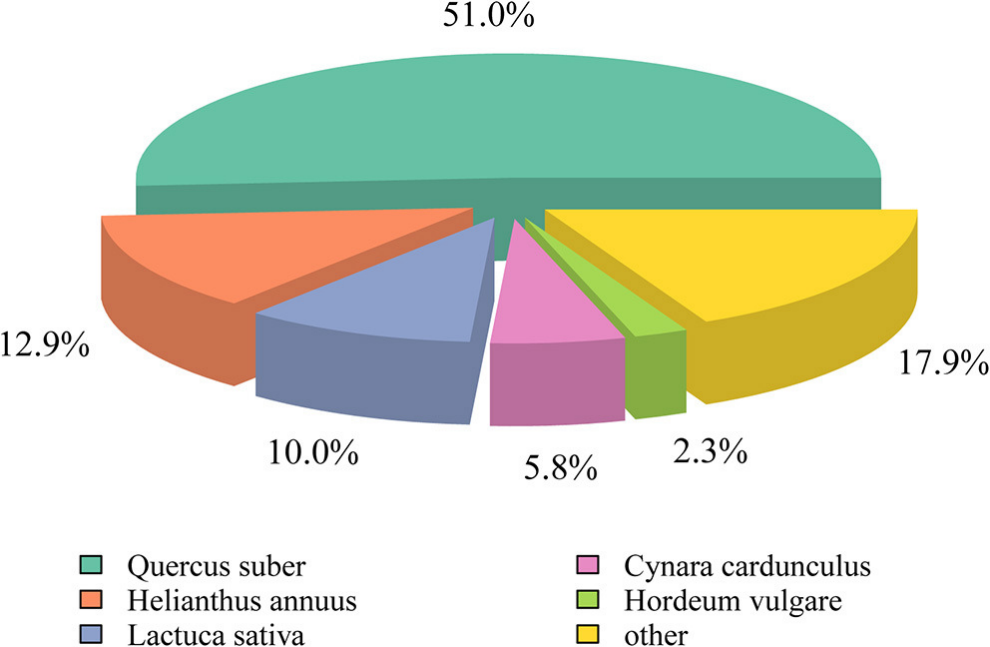
The percentage (%) of *C. rhombifolium* unigenes hits the species in the Nr database.

We used the Nr, Nt, Pfam, KOG, Swiss-prot, GO, and KEGG databases to annotate the assembled unigenes. Among the 130,891 unigenes obtained, at least 97,496 unigenes (74.48%) could be annotated with the searched databases −40,878 (Nr), 55,831 (Nt), 37,488 (KO), 74,084 (Swiss-prot), 56,213 (Pfam), 56,213 (GO), and 42,005 (KOG/COG), suggesting that this project generated a substantial fraction of the expressed genes in this study (Table 2). The unigenes annotated with the Nr database mainly comprised *Quercus suber* L. (51%), *Helianthus annuus* L. (12.9%), *Lactuca sativa* Linn. (10%), *Cynara cardunculus* L., Sp. Pl. (5.8%), and *Hordeum vulgare* Linn. (2.3%) sequences (Figure 2). The highest similarity observed was with *Q. suber*, a species with resistance to wind, drought, and barren environments (Pereira-Leal et al., 2014).

**Table 2:** Summary of functional annotation of assembled unigenes in *C. rhombifolium*.

**Database**	**Number of unigenes annotated**	**Percentage (%)**
Annotated in NR	40,878	31.23
Annotated in NT	55,831	42.65
Annotated in KO	37,488	28.64
Annotated in SwissProt	74,084	56.59
Annotated in PFAM	56,213	42.94
Annotated in GO	56,213	42.94
Annotated in KOG	42,005	32.09
Annotated in all databases	7,286	5.56
Annotated in at least one database	97,496	74.48
Total unigenes	130,891	100

**Figure 3:**
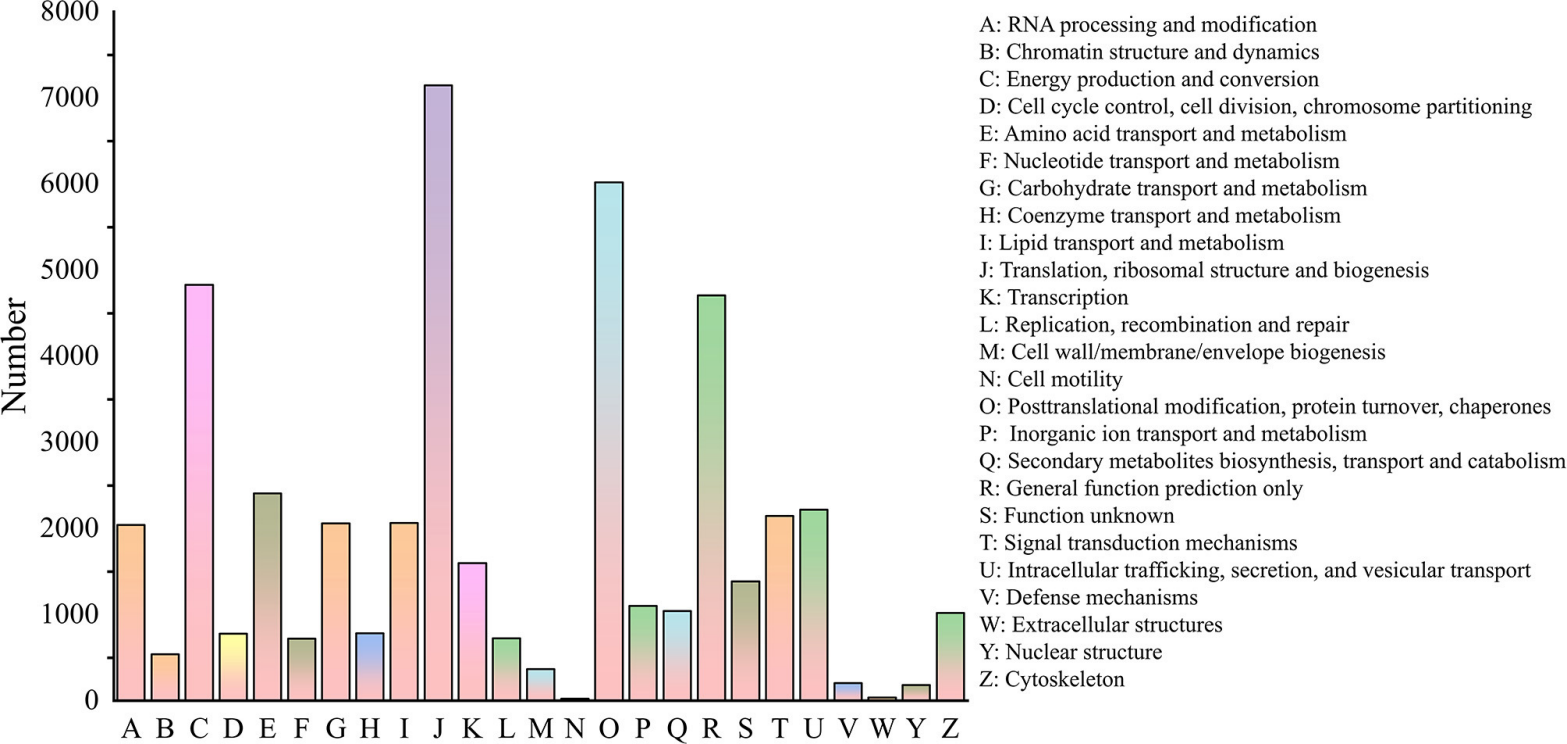
COG classifications of unigenes in the transcriptome of *C. rhombifolium* unigenes.

The COG analysis enabled the functional classification of 42,005 unigenes (Figure 3). The most frequently identified classes were “Translation, ribosomal structure and biogenesis” (7,147; 17%), followed by “Posttranslational modification, protein turnover, chaperones” (6,016; 14.3%), “Energy production and conversion” (4,830; 11.5 %), “General function prediction only” (4,706; 11.2%), “Amino acid transport and dynamics” (2407; 5.7%), “Intracellular trafficking, secretion, and vesicular transport (2218; 5.3%), “signal transduction” (2,145; 5.1%), “Lipid transport and metabolism” (2065, 4.9%) and “Carbohydrate transport and metabolism (2057; 4.9%). The least frequently identified groups were “Nuclear structure” (183; 0.4%), “Extracellular structures” (36; 0.09%), and “Cell motility” (24; 0.06%). Similar patterns have been reported in some angiosperms, such as *Chrysanthemum nankingense* (Wang et al., 2013) and *Camelina sativa* (Liang et al., 2013). We found 205 unigenes belonging to “Defense mechanism,” which indicated the existence of stress resistance genes in *C. rhombifolium*.

**Figure 4:**
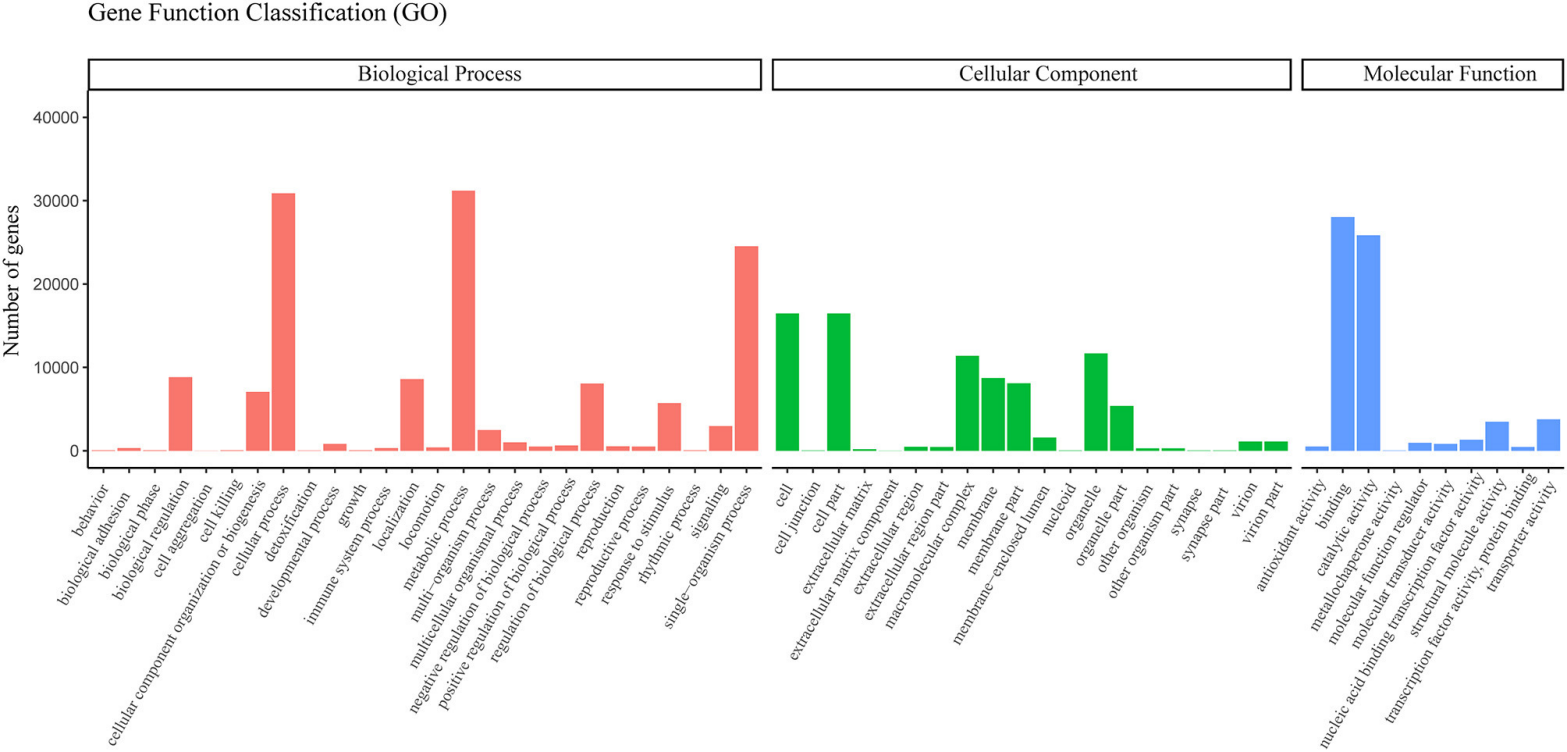
Functional classification of *C. rhombifolium* unigenes based on Gene Ontology (GO) categorization. The results are summarized in three main GO categories: biological process (shown in red color), cellular component (shown in green color) and molecular function (shown in blue color). The x-axis indicates the subcategories, and the y-axis indicates the numbers related to the total number of GO terms present.

Based on the Nr annotation, 42,005 unigenes were assigned to three ontologies and classified into 48 functional GO categories using the Blast2GO software. Of these, 1050 (68.5%), 163 (10.6%), and 320 (20.9%) GO terms were related to cellular components, biological processes, and molecular functions, respectively (Figure 4). The assignment of GO terms in *C. rhombifolium* in this study focused on “cellular processes,” “metabolic processes,” “single-organism processes,” “cell,” “cell parts,” “macromolecular complex,” “membrane part,” “organelles,” and “binding and catalytic activity,” which reflected the functional gene expression characteristics during its normal growth. This result was similar to those GO terms in some drought- resistance species, e.g., bread wheat, oak, *Boea hygrometrica, Boea hygrometrica*, and so on (Gupta et al., 2003; Durand et al., 2010; Xiao et al., 2015; Zhu et al., 2015), which mainly due to the selective gene expression caused by various environments and physiological states.

**Figure 5:**
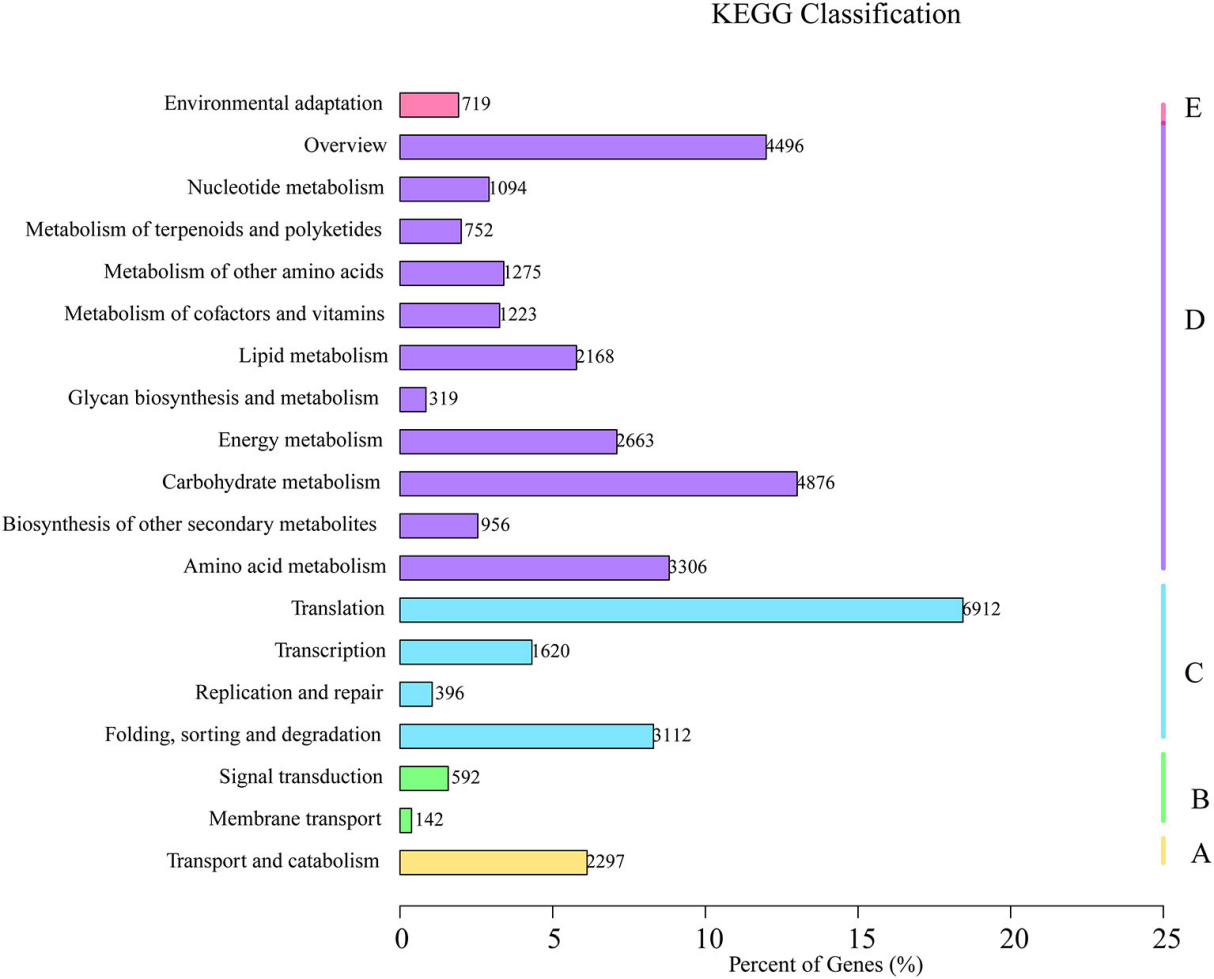
Pathway assignment based on the Kyoto Encyclopedia of Genes and Genomes (KEGG). **(A)** Classification based on cellular process categories, **(B)** classification based on environmental information processing categories, **(C)** classification based on genetic information processing categories, **(D)** classification based on metabolism categories, and **(E)** classification based on organismal systems categories.

Based on the sequence homology searches against the KEGG database, 56,213 unigenes were assigned to five ontologies and classified into 18 functional KEGG pathways (Figure 5). Among these pathways, the “translation pathway” (6,912; 12.3% of KEGG unigenes) was the largest category in “metabolism” followed by “carbohydrate metabolism” (4,876), “overview” (4,496), “amino acid metabolites” (3,306), “folding, sorting, and degradation” (3,112), “energy metabolism” (2,663), “transport and catabolism” (2,297), and “lipid metabolism” (2,168). In this study, we highlight the pathways enriched for the interaction between plants and their environment, including: “metabolism of terpenoids and polyketides” (752), “signal transduction” (592), “environmental adaptation” (719), and “replication and repair” (396). Our results are consistent with those from other studies identifying plant genes and gene products with important roles in drought-resistant plants (Gechev et al., 2012; Xiao et al., 2015). More than 74% of the unigenes from *C. rhombifolium* were mapped in the known databases, which is higher than that reported for *C. nankingense* (64%) (Wang et al., 2013).

### Frequency and Distribution of SSRs

**Figure 6:**
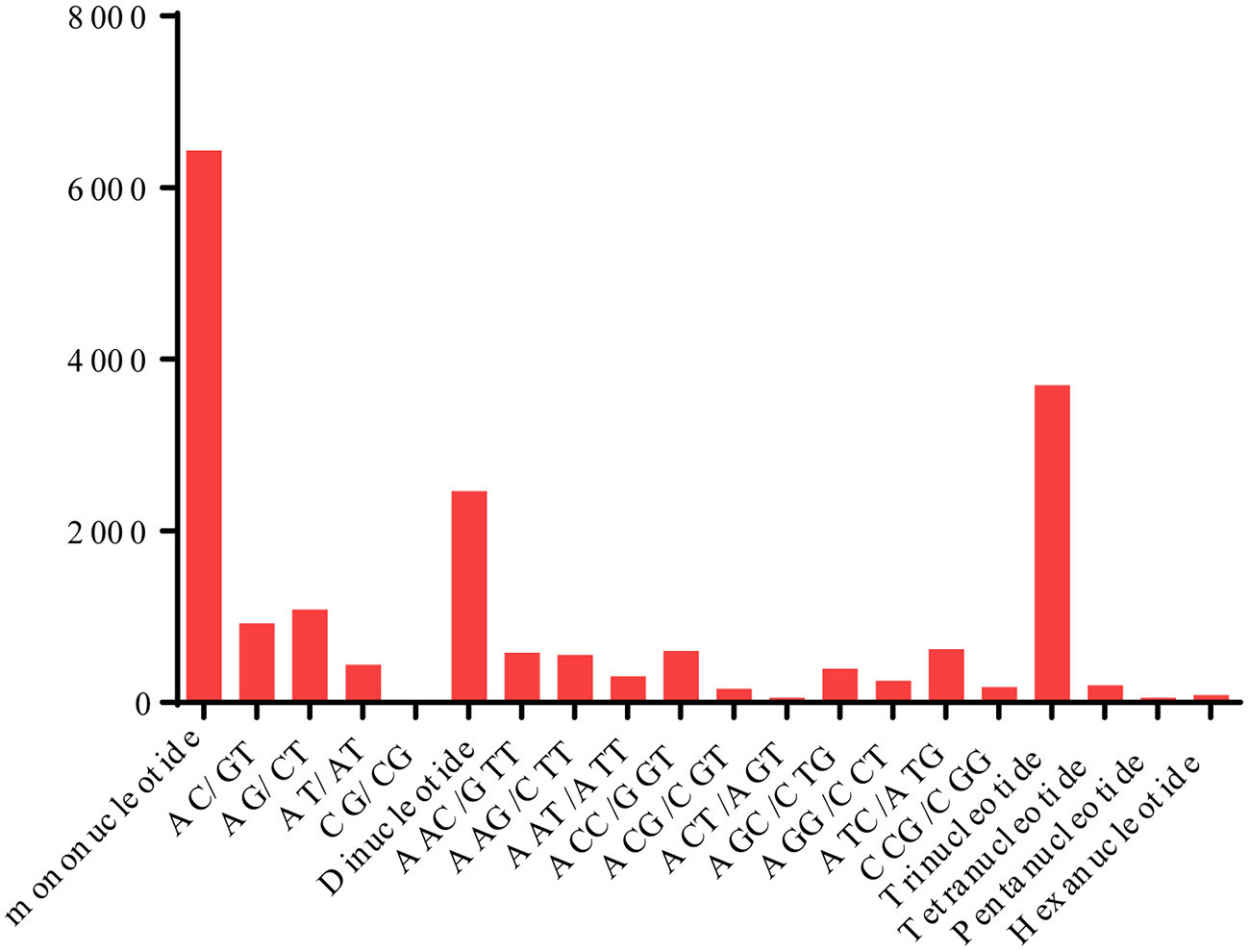
Simple sequence repeat (SSR) types present in the *C. rhombifolium* transcriptome.

In total, 12,925 SSR regions were identified in 10,524 unigenes. Among the identified SSRs, 128 different motifs were identified, the distribution and frequencies of which are shown in Figure 6. Mononucleotide motifs were the most abundant, and A/T were the largest subset (6,328). Overall, 6,429 mononucleotide, 2,463 di-repeats, 3,694 tri-repeats, 199 tetra-repeats, 56 penta-repeats, and 84 hexa-repeats were found in the *C. rhombifolium* leaf transcriptome. Among the unigenes containing SSRs, 941 SSRs presented compound formation, and 1,874 contained more than one SSR. On average, one SSR was found every 8.17 kb. The observed number of SSR sequences in our study was higher than EST-SSR ever reports in *Chrysanthemum* (Wang et al., 2013). The SSR sequences may gain or loss of repeats at a locus in their rapid evolution for adaptation to various environments (King et al., 1997; Trifonov, 2004). The mass EST-SSR loci in *C. rhombifolium* may be caused by its harsh habitats. These ESTs will provide a valuable repository of abundant information for future functional SSR studies.

### Phylogenetic Analysis of GME

**Figure 7:**
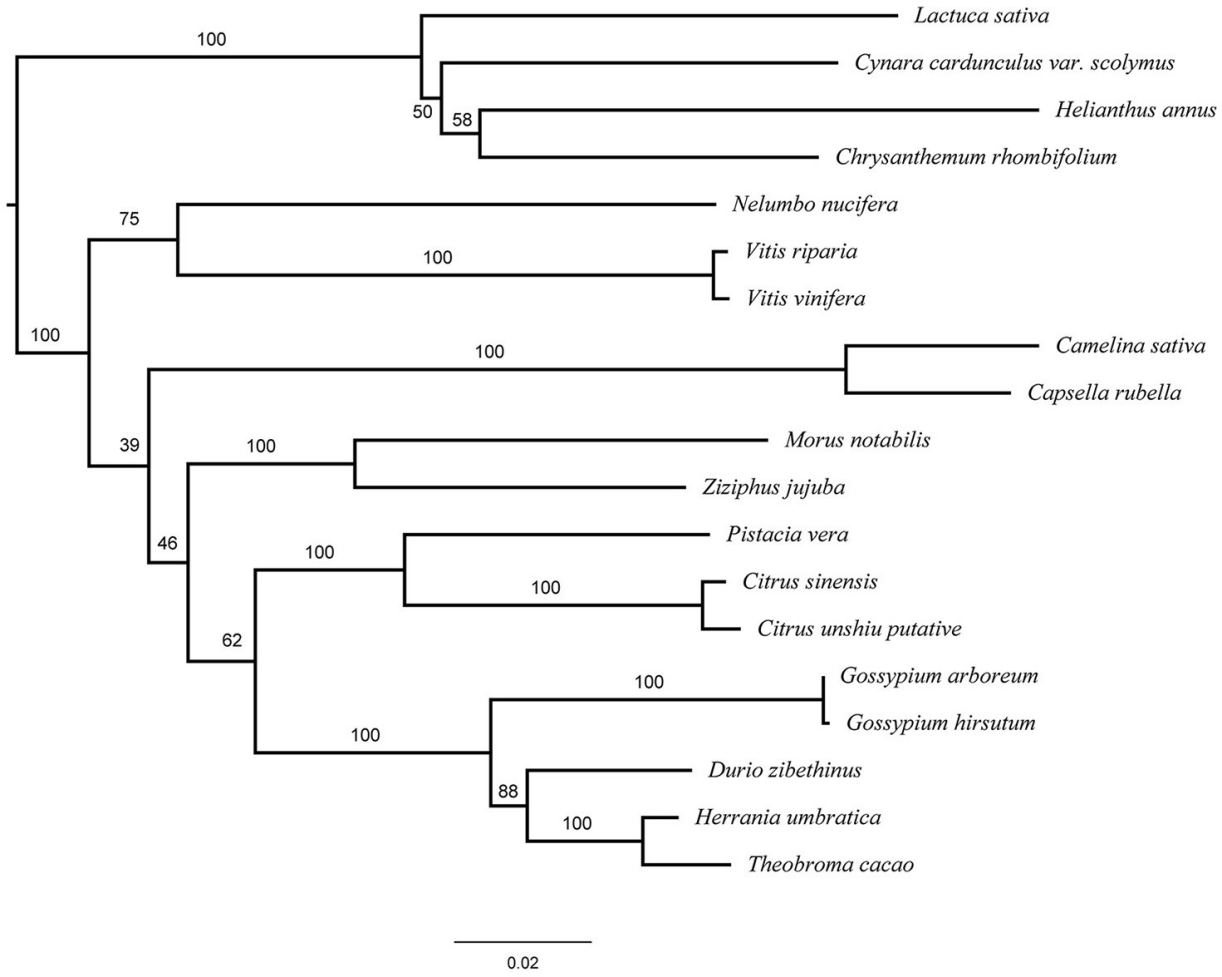
Phylogenetic analysis of the GME in plants. *Helianthus annuus* XM_022174384.1; *Lactuca sativa* (XM_023912155.1); *Cynara cardunculus* var. scolymus (XM_025140401.1), *Chrysanthemum rhombifolium* (R|Cluster-20886.25690); *Camelina sativa* (XM_010456910.1); *Capsella rubella* (XM_006287892.2); *Nelumbo nucifera* (XM_010275940.2); *Morus notabilis* (XM_024169380.1); *Vitis riparia* (XM_034830226.1); *Vitis vinifera* (XM_002283862.4); *Ziziphus jujuba* (XM_016019918.2); *Gossypium arboretum* (XM_017764784.1); *Gossypium hirsutum* (XM_016869694.1); *Durio zibethinus* (XM_022891507.1); PREDICTED: *Herrania umbratica* (XM_021427500.1); *Theobroma cacao* (XM_018122066.1); *Pistacia vera* (XM_031412928.1); *Citrus sinensis* (XM_006471610.2); *Citrus unshiu* (HQ224947.1).

Using the annotated sequence of *GME* in *C. rhombifolium* and other *GME* homologs, we constructed a phylogenetic tree among related species. All of the *GME* sequences from the same taxa were clustered together and *GME* in *C. rhombifolium* were grouped into a single clade with the sequences of *Helianthus annuus* and other Asteraceae species (Figure 7), this result revealed a close relationship of *C. rhombifolium* and other Asteraceas species, which consistent with the taxonomy based on morphology.

## Conclusions

We obtained 130,891 unigenes from the leaf of *C. rhombifolium* by NGS transcriptomics, of which 97,496 (~74.5%) unigenes were successfully annotated in the public protein database. A total of 12,925 SSRs were detected in 10,524 unigenes. This is the first genetic study of *C. rhombifolium* as a plant resource of stress-tolerant genes. These large numbers of transcriptome sequences have enabled us to comprehensively understand the basic genetics of *C. rhombifolium* and discover novel genes that will be helpful in the molecular improvement of chrysanthemums.

## Data Availability Statement

The datasets presented in this study can be found in GenBank. The accession numbers can be found in the article.

## Author Contributions

YM conceived and designed the experiments. LZ collected the plants. WZ, HX, XD, JL, and JH performed the experiments and analyzed the data. YM, WZ, and LZ wrote the paper. All authors contributed to the article and approved the submitted version.

## Conflict of Interest

The authors declare that the research was conducted in the absence of any commercial or financial relationships that could be construed as a potential conflict of interest.
